# Stock-outs of antiretroviral and tuberculosis medicines in South Africa: A national cross-sectional survey

**DOI:** 10.1371/journal.pone.0212405

**Published:** 2019-03-12

**Authors:** Bella Hwang, Amir Shroufi, Tinne Gils, Sarah Jane Steele, Anna Grimsrud, Andrew Boulle, Anele Yawa, Sasha Stevenson, Lauren Jankelowitz, Marije Versteeg-Mojanaga, Indira Govender, John Stephens, Julia Hill, Kristal Duncan, Gilles van Cutsem

**Affiliations:** 1 Médecins Sans Frontières South Africa, Operational Control Centre Brussels, Cape Town, South Africa; 2 International Aids Society, Geneva, Switzerland; 3 Centre for Infectious Disease Epidemiology and Research, University of Cape Town, Cape Town, South Africa; 4 Treatment Action Campaign, Johannesburg, South Africa; 5 SECTION27, Johannesburg, South Africa; 6 Southern African Clinicians Society, Johannesburg, South Africa; 7 Rural Health Advocacy Project, Johannesburg, South Africa; 8 Rural Doctors Association of Southern Africa, Johannesburg, South Africa; 9 Médecins Sans Frontières, Southern African Medical Unit, Cape Town, South Africa; The Ohio State University, UNITED STATES

## Abstract

**Background:**

HIV and TB programs have rapidly scaled-up over the past decade in Sub-Saharan Africa and uninterrupted supplies of those medicines are critical to their success. However, estimates of stock-outs are largely unknown. This survey aimed to estimate the extent of stock-outs of antiretroviral and TB medicines in public health facilities across South Africa, which has the world’s largest antiretroviral treatment (ART) program and a rising multidrug-resistant TB epidemic.

**Methods:**

We conducted a cross-sectional telephonic survey (October—December 2015) of public health facilities. Facilities were asked about the prevalence of stock-outs on the day of the survey and in the preceding three months, their duration and impact.

**Results:**

Nationwide, of 3547 eligible health facilities, 79% (2804) could be reached telephonically. 88% (2463) participated and 4% (93) were excluded as they did not provide ART or TB treatment. Of the 2370 included facilities, 20% (485) reported a stock-out of at least 1 ARV and/or TB-related medicine on the day of contact and 36% (864) during the three months prior to contact, ranging from 74% (163/220) of health facilities in Mpumalanga to 12% (32/261) in the Western Cape province. These 864 facilities reported 1475 individual stock-outs, with one to fourteen different medicines out of stock per facility. Information on impact was provided in 98% (1449/1475) of stock-outs: 25% (366) resulted in a high impact outcome, where patients left the facility without medicine or were provided with an incomplete regimen. Of the 757 stock-outs that were resolved 70% (527) lasted longer than one month.

**Interpretation:**

There was a high prevalence of stock-outs nationwide. Large interprovincial differences in stock-out occurrence, duration, and impact suggest differences in provincial ability to prevent, mitigate and cope within the same framework. End-user monitoring of the supply chain by patients and civil society has the potential to increase transparency and complement public sector monitoring systems.

## Introduction

In recent years, health programs in Sub-Saharan Africa rapidly scaled up access to HIV and tuberculosis (TB) treatment. South Africa has the highest number of people living with HIV, the highest incidence of TB worldwide, a rising epidemic of multidrug resistant (MDR) TB and the world’s largest antiretroviral treatment (ART) program [1]. Expansion of the eligibility threshold for ART has increased demand on the under-resourced health system, with 3.9 of 7.1 million people living with HIV (PLHIV) on ART in 2016 [2]. Uninterrupted supply of antiretroviral (ARV) and TB medicines is critical to controlling the HIV and TB epidemics [3, 4]. In a context of increasing strain on a fragile health system with limited resources, the risk associated with stock-outs is significant and requires enhanced attention, monitoring and strengthening of the supply chain [5].

Stock-outs are defined by the WHO as the complete absence of a required medicine for at least one day at a storage or delivery point [6]. A stock-out of any ARV routinely used, is an early warning indicator for the development of ART resistance [7]. Health-care worker coping strategies such as shortening of the refill period, borrowing medicine or referring patients to other facilities [8] increase patient-borne costs and can lead to unplanned treatment interruptions [9]. Such unplanned interruptions are associated with an increased risk for opportunistic infections, virologic failure and drug resistance [9, 10]. Patients may also experience a significant financial burden [11] due to additional transport costs and experience an increase in time to access treatment, potentially discouraging patients from optimal adherence. In addition, ARV stock-outs have a negative impact on retention in care and patient survival [12, 13]. A study in South Africa concluded that patients who claimed less than 80% of their prescription refills were three times more likely to die than those who claimed 80% or more [14].

In South Africa, stock-outs have been reported throughout the years [15–19]. The National Department of Health (NDoH) has identified medicine stock-outs as a major health systems concern [20]. However, the current monitoring system does not provide transparent information on medicine availability and the true extent of the problem across the country is not known. Despite ongoing public reports of stock-outs, a 2013 WHO Joint Review of HIV, TB, and PMTCT Programmes in South Africa reported that stock-outs of ART in the previous 12 months were minimal [21]. This survey provides country-level estimates of the extent, impact and duration of stock-outs of ARV and TB medicines in South Africa.

## Methods

### Overview of study design

We conducted a cross-sectional telephonic survey of all health facilities in the South African public sector, identified from the national District Health Information Database (DHIS 2), between October 1^st^ and December 11^th^ 2015. At each facility, one staff member was invited to respond in the following order of preference: pharmacist, pharmacy assistant, person who orders the medicine, nurse-in-charge or nurse. Participants provided verbal informed consent. Risk to participants was minimized by anonymizing participant and facility name, and aggregating results to the provincial and national levels.

### Measurement

Data was collected through a previously piloted standard questionnaire by trained research assistants and recorded in an electronic database. Participants were asked to provide the name of each ARV and TB-related medicine that was out of stock on the day of contact and any additional stock-out experienced by the facility in the three-month period prior to contact. Respondents were asked to report (i) the duration of stock-outs, (ii) the resulting action of the facility and (iii) the quantity of medicines the patient received as compared to standard of care. Responses were subsequently categorized (Table 1). Duration of stock-outs was only reported if any supply of medicine was delivered to the facility after the stock-out was reported.

**Table 1:** Stock-out attributes categories.

Variable	Measurement (categorical variable)
**Stock-out duration**	• Less than 1 week • 1–4 weeks • More than 4 weeks
**Facility reaction to stock-out**	• Referred patients OR turned patients away • Dispensed only part of the formulations constituting a full regimen • Borrowed from another facility • Substituted by the same regimen but at a less optimal dosage OR a less optimal formulation OR pill burden increased* • Switched to a less optimal regimen* • Switched appropriately to a different regimen, dosage or formulation*
**Quantity of medicine dispensed to patient**	• No medication at all • A smaller supply • A full supply

* Regimens were assessed to contain only part of the formulations constituting a full regimen, and switches were determined as less optimal or appropriate, according to regimen choices outlined in the national guidelines.

A “stock-out” was defined as the complete absence of a specific formulation and/or dosage of medicine at a given facility. Stock-outs “on the day of contact” were defined as occurring on the day the facility responded. Those “occurring in the three-month period prior to contact” were defined as occurring on the day of contact or the ninety days prior. Each stock-out was classified as having a high, medium or low impact on patients based on the action of the facility and the supply given to the patient (Table 2). A site was defined as having a stock-out if at least one ARV and/or TB medicine stock-out was reported. Some facilities reported more than one medicine out of stock and this frequency is reported. Data were stratified into four medicine types according to the South African National Guidelines: ARVs for adults, nevirapine (NVP) syrup for the prevention of mother-to-child transmission of HIV (PMTCT), paediatric ARVs and TB-related medicines. Adult ARVs were further stratified into first-line, second line and less commonly used ARVs (Appendix A) as recommended by the National Guidelines at the time of the study.

**Table 2:** Definitions of high, medium and low impact of stock-outs.

Impact category*	Facility action	Quantity of medicine dispensed to patient
High	Patients referred elsewhere OR turned away	No medication
	Dispensed only part of the formulations constituting a full regimen	A smaller OR full supply
Medium	Patients referred elsewhere OR turned away	A smaller supply
	Borrowed from another facility	A smaller supply
	Substituted by the same regimen but a less optimal dosage OR a less optimal formulation OR pill burden increased	A smaller OR full supply
	Switched to a less optimal regimen	A smaller OR full supply
Low	Switched appropriately to a different regimen, dosage or formulation	A full supply
	Borrowed from another facility	A full supply

*Both facility action and quantity of medicine dispensed to patient need to be fulfilled to meet the definition

### Validation of measures

We conducted a validation sub-study to determine the reliability of the measures in a random sample of facilities (n = 159). These facilities were surveyed twice on the same day, with two different participants providing answers to the same questions. Specifically, both participants were asked “Today do you have an ARV or TB medicine unavailable?” When it was not possible to reach a second participant on the same day, the second participant was surveyed on the following day.

### Statistical analysis

Statistical analyses were performed using Stata 13.0. Means and medians were calculated using respondents for the question of interest as the denominator. We conducted standard descriptive statistics to compare across strata of interest. We used t-tests to compare continuous variables, and either chi-square or Fisher’s exact tests to compare categorical variables. We assessed ordinal variables using Wilcoxon Rank-Sum test. For the purpose of validating our measures we used the kappa coefficient as a measure of agreement between the responses by the first and second participant from validation sites.

### Ethics

Ethical approval was granted from the Human Research Ethics Committee of the University of Cape Town (624/2015) and the Ethics Review Board of Médecins Sans Frontières (1548). As this was a telephonic study, no written consent was obtained. Facility representatives were asked to provide oral informed consent to participate as a respondent before answering the survey questionnaire which was manually recorded in the electronic database. The verbal consent procedure was approved by both ethical boards.

## Results

**Fig 1:**
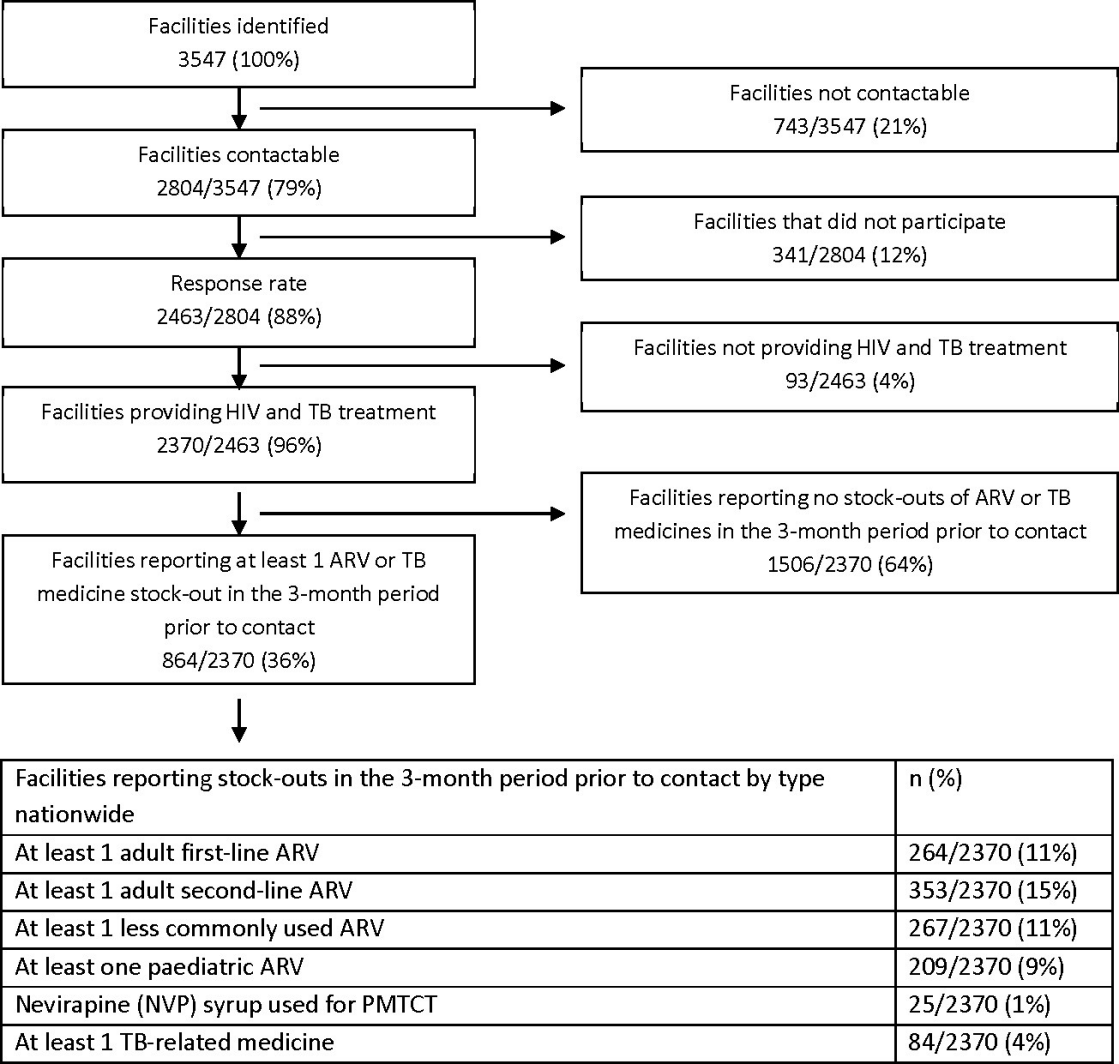
Study overview: Flow diagram of study population and responses.

**Fig 2:**
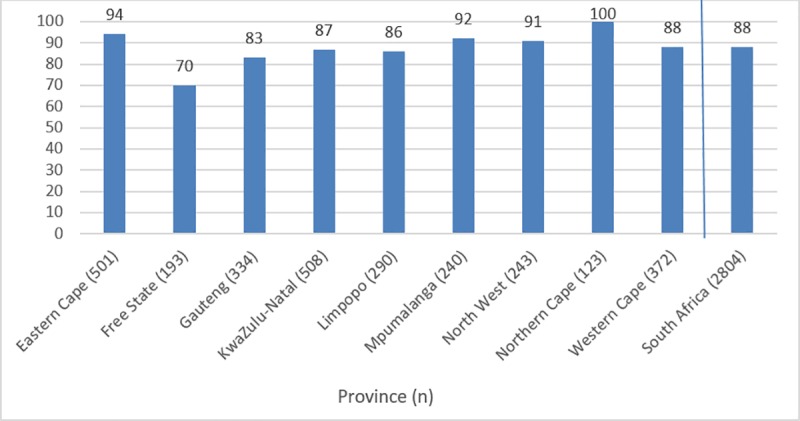
Percentage of facilities responding to survey by province and nationwide.

A total of 3547 facilities were identified as eligible for inclusion; 79% (2804) could be reached telephonically, of which 88% (2463) participated in the study; 4% (93) did not provide ARV or TB treatment and are not included in this analysis (Figs 1 and 2). 98% (2423) of participants provided information about their position: 58% (1396) were nurses-in-charge, 17% (408) nurses, 16% (392) pharmacists and 9% (227) pharmacy assistants.

**Fig 3:**
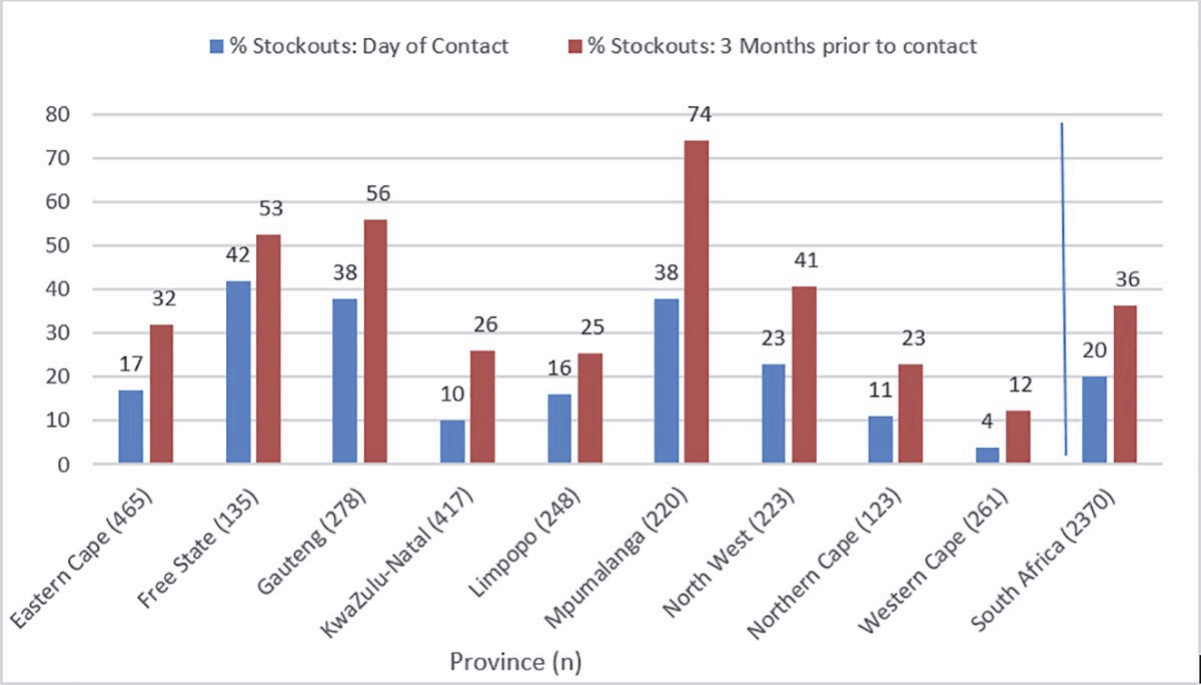
Percentage of facilities reporting at least one ARV or TB medicine stock-out on the day of contact and in the 3 months prior to contact by province and nationwide.

Nationwide, 20% (485/2370) of facilities reported a stock-out on the day of contact and 36% (864/2370) reported at least 1 ARV and/or TB-related medicines stock-out during the three months prior to contact, ranging from 74% (163/220) in Mpumalanga province to 12% (32/261) in the Western Cape province (Fig 3).

In the three months prior to contact, there was a stock-out of at least one: adult first-line ARV in 11% (264/2370), adult second-line ARV in 15% (353/2370), less commonly used ARV in 11% (267/2370), pediatric ARV in 9% (209/2370), TB-related medicines in 4% (84/2370) and nevirapine syrup in 1% (25/2370) of facilities (Fig 3).

**Fig 4:**
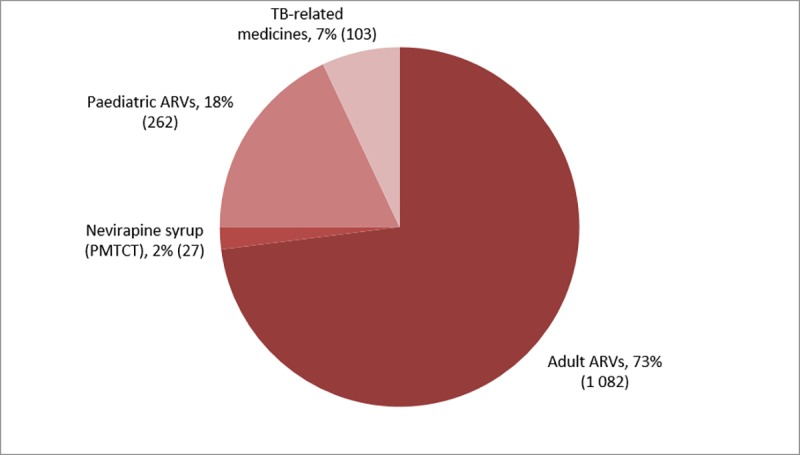
Breakdown by type of medicine of stock-outs of ARVs and TB-related medicines reported nationwide.

A total of 1475 single medicine stock-outs were reported (Table 3) across 36% (864/2370) facilities, with one to fourteen different medicines out of stock per facility. Of these 1475 stock-outs, 73% (1082) were of ARVs for adults; 18% (262) were of paediatric ARVs, 2% (27) were of NVP syrup and 7% (103) were of TB-related medicines (Fig 4 and Table 4).

**Table 3:** Medicines reported out of stock during the three months prior to contact in South African facilities.

Name and formulation of medicine	Number of reported stock-outs (%)*	Name and formulation of medicine	Number of reported stock-outs (%)*
(LPV/r) Lopinavir/Ritonavir 200mg/50mg, tablet	348 (24%)	(EFV) Efavirenz 50mg, tablet	14 (1%)
(ABC) Abacavir 600mg, tablet	278 (19%)	(R/H) Rifampicin/Isoniazid 300/150mg, tablet	14 (1%)
(3TC) Lamivudine 150 or 300 mg, tablet	197 (13%)	(RHZE) Rifampicin/Isoniazid /Pyrazinamide/Ethambutol 150/75/400/275mg, tablet	12 (0.8%)
(AZT) Zidovudine 300mg, tablet	72 (5%)	(TDF/FTC) Tenofovir/ Emtricitabine 300/200mg, tablet	9 (0.6%)
(ABC) Abacavir 60mg tablet or 20mg/ml solution	71 (5%)	(R/H) Rifampicine/Isoniazid 60/60mg, tablet	9 (0.6%)
TDF/FTC/EFV Tenofovir/Emtricitabine/Efavirenz 300/200/600mg, tablet	62 (4%)	(RTV) Ritonavir 100mg, tablet	8 (0.5%)
(LPV/r) Lopinavir/Ritonavir 80/20mg/ml, solution	57 (4%)	(D4T) Stavudine 30mg, tablet	7 (0.5%)
(LPV/r) Lopinavir/Ritonavir 100/25mg, tablet	36 (2%)	(D4T) Stavudine 15mg or 20 mg, tablet	7 (0.5%)
(EFV) Efavirenz 200mg, tablet	30 (2%)	(ABC/3TC) Abacavir/Lamivudine 600/300mg, tablet	6 (0.4%)
(NVP) Nevirapine 50mg/50ml, solution	27 (2%)	(AZT) Zidovudine solution 50mg/5ml	6 (0.4%)
(EFV) Efavirenz 600mg, tablet	26 (2%)	(E) Ethambutol 400mg, tablet	5 (0.3%)
(NVP) Nevirapine 200mg, tablet	26 (2%)	(R) Rifampicin 300mg, capsule	5 (0.3%)
(DDi) Didanosine, tablet	21 (1%)	(PZA) Pyrazinamide 150mg, tablet	4 (0.3%)
(3TC) Lamivudine 10mg/ml solution	21 (1%)	(PZA) Pyrazinamide 500mg, tablet	4 (0.3%)
(INH) Isoniazid 300mg, tablet	21 (1%)	(Lvx) Levofloxacin 250 or 500mg, tablet	4 (0.3%)
(R/H) Rifampicin/Isoniazid 150/75mg, tablet	18 (1%)	(RTV) Ritonavir 80mg/ml, solution	3 (0.2%)
(TDF) Tenofovir 300mg, tablet	15 (1%)	Other	10 (0.7%)
(AZT) Zidovudine 100mg, tablet	14 (1%)		

^*^ Percentages are number of reported stock-outs for individual medicine divided by total number of reported stock-outs (1475)

**Table 4:** Percentage of facilities reporting stock-outs with one, two or three or more stock-outs reported on the day of contact and in the three months prior to contact.

Survey period (N)	% One stock-out (n)	% Two stock-outs (n)	% Three or more stock-outs (n)
On the day of contact (485)	70% (339)	20% (97)	10% (49)
Three months prior to contact (864)	61% (530)	23% (201)	15% (133)

Of 1082 stock-outs of adult ARVs, 29% (315) were first-line ARVs, 41% (449) second-line ARVs and 29% (315) were less commonly used ARVs. The main driver of second-line ARV stock-outs was a national shortage of adult LPV/r making up 77% (348/449) of second-line adult ARV stock-outs and accounting for 24% (348/1475) of stock-outs overall. Stock-outs of adult abacavir (ABC) were reported in 19% (278) of instances. ABC could have been used to substitute for LPV/r in some cases during shortages, and contributed to stock-outs of less commonly used ARVs.

**Fig 5:**
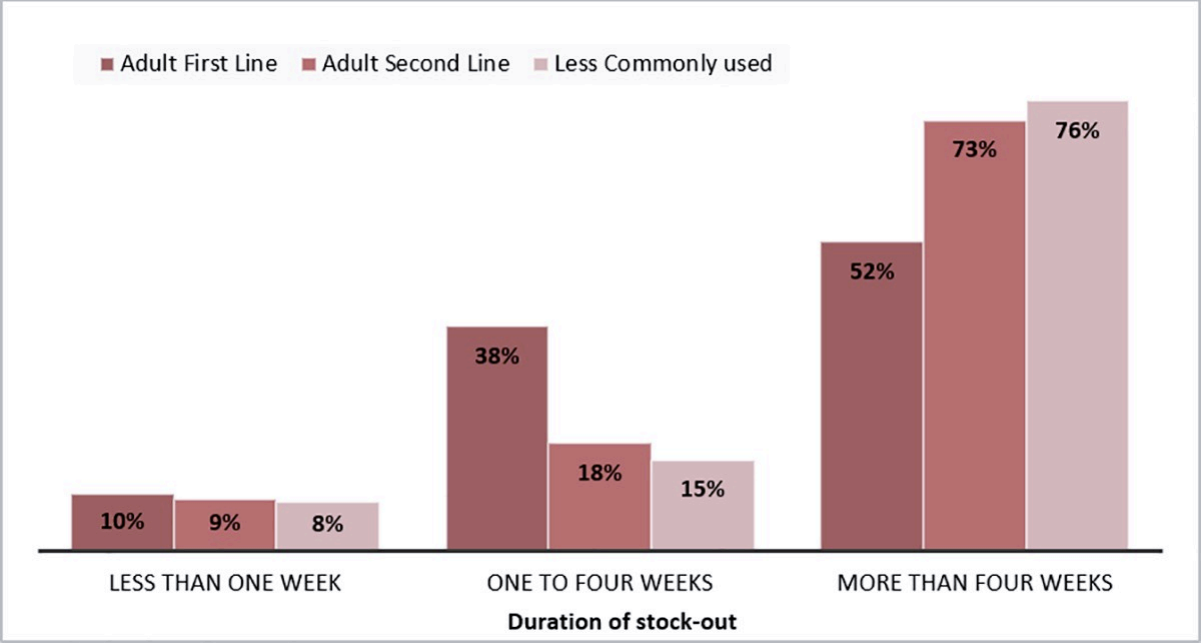
Percentage of resolved stock-outs lasting (i) less than 1 week, (ii)1-4 weeks, and (iii) more than 1 month by type of ARV.

**Fig 6:**
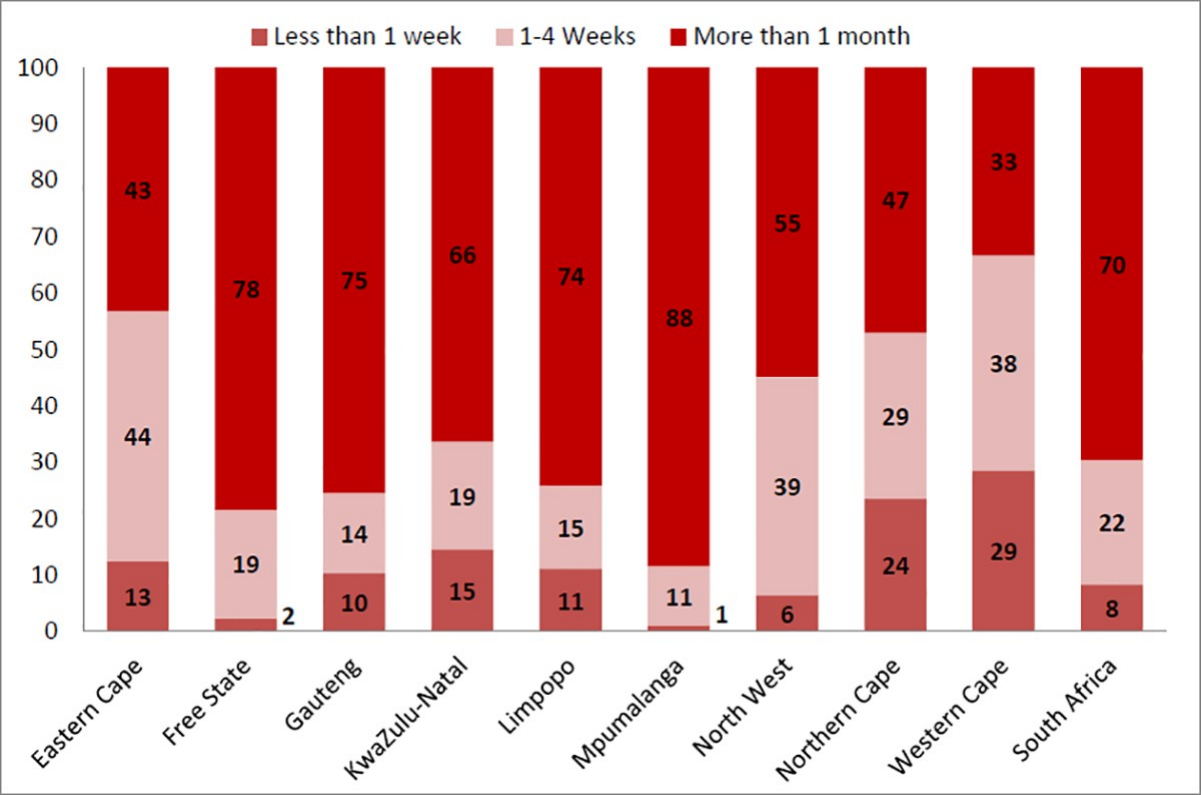
Percentage of resolved stock-outs lasting (i) less than 1 week, (ii) 1–4 weeks, and (iii) more than 1 month by type of province.

Respondents reported that of 757 stock-outs that had been resolved, 8% (63) lasted less than a week, 22% (167) between one and four weeks and 70% (527) longer than one month (Fig 5). There was a wide variation in the time to resolve stock-outs between provinces (Fig 6).

Information on impact of the stock-out was provided in 98% (1449/1475) of stock-outs. In 25% (366/1449) of cases, patients left the facility without any treatment or were provided with an incomplete treatment regimen (high impact); 39% (561/1449) had a medium impact and 36% (522/1449) had a low impact (Table 5).

**Table 5:** Proportions of stock-outs leading to high, medium, and low impact out of 1449 individual stock-outs reported by province and nationwide in the three-month period prior to contact.

Province (N)	% High impact stock-out (n)	% Medium impact stock-out (n)	% Low impact stock-out (n)
Eastern Cape (180)	20% (36)	18% (32)	62% (112)
Free State (210)	38% (80)	20% (42)	42% (88)
Gauteng (268)	17% (45)	59% (157)	25% (66)
KwaZulu-Natal (167)	14% (24)	68% (114)	17% (29)
Limpopo (73)	32% (23)	22% (16)	47% (34)
Mpumalanga (322)	28% (91)	33% (106)	39% (125)
North West (167)	32% (54)	47% (78)	21% (35)
Northern Cape (33)	27% (9)	6% (2)	67% (22)
Western Cape (29)	14% (4)	48% (14)	38% (11)
South Africa (1449)	25% (366)	39% (561)	36% (522)

N is number of stock-outs in the province; % is percentage of total stock-outs in the province by impact category.

Of 2370 facilities nationwide, 10% (242) reported at least 1 high impact, 15% (366) at least 1 medium impact and 16% (374) at least one low impact stock-out.

Of the 159/2463 (7%) facilities randomly selected for the validation study, 90% (143/159) responded. Validation participants provided the same answer as the primary participant 94% of the time, suggesting near perfect agreement between the results reported by the different participants (Kappa coefficient = 0.84)

## Discussion

This is, to our knowledge, the first national stock-out survey conducted in a resource-limited setting. Results show that stock-outs of HIV and TB medicines were widespread in South Africa. Nationally, more than one third of health facilities had a stock-out of ARV and/or TB medicines in the three months preceding the survey; a quarter of these stock-outs had a high impact on patients, who left the facility without or with an insufficient quantity of medicines. One fifth of facilities had a stock-out on the day they were contacted.

A national stock-out of adult LPV/r caused one quarter of facility stock-outs, particularly contributing to stock-outs in the provinces of Gauteng and Free State. Supply fragility due to a single international supplier contributed to this specific stock-out. All other HIV and TB-related medicines reported out of stock were available in-country, indicating downstream deficiencies.

Regardless of the cause, large interprovincial differences remained in stock-out occurrence, duration and impact, suggesting differences in provincial ability to prevent, mitigate and cope within the same national framework. The long duration of existing stock-outs indicates the lack of end-level visibility and mitigation mechanisms to alert and solve stock-outs when they occur. A national telephonic survey can be used as an inexpensive tool to rapidly assess supply in facilities or audit existing monitoring systems.

Consequences of ART and TB stock-outs are ultimately borne by patients, putting them at risk of treatment interruption or discontinuation, catastrophic expenditure [14,15], treatment failure and drug resistance [21], and ultimately increased risk of illness and death [12,22]. In 25% of reported stock-outs patients were either turned away without medication or given an incomplete regimen.

Stock-outs of adult ARVs were most common (74%), fueled by stock-outs of LPV/r and ARVs used for its substitution. Only 4% of stock-outs were of the fixed-dose combination (FDC) of TDF/3TC/EFV used as first line ART for the majority of patients on HIV treatment. Stock-outs were more frequent of alternative ARVs used for a smaller group of more vulnerable patients, such as those with contraindications or drug resistance.

Stock-outs are reported to be widespread in other countries in sub-Saharan Africa, with proportion of months with ART stock-outs higher in Africa than other continents. Data from WHO show that in 1703 clinics in 35 countries, 35.7% had an ART stock-out over a one-year period between 2005 and 2014 [23]. Clinics with more stock-outs also had worse on-time appointment keeping, on time pill pick-up and retention in care [23]. A 2015 study from Kinshasa found that stock-outs of HIV commodities were common especially due to downstream problems in the last part of the chain [24]. Over half of ART stock-out cases resulted in patients leaving the facility without ARVs [24]. A study of 20 health centers in Ethiopia revealed that 73.7% had a stock-out of one or more ARV medicines on the day of visit; contributing factors included inaccurate use of the stock management software and inappropriate patient data collection, limiting supply planning capacity [25]. A similar study in Lesotho proposed that a lack of supervisory visits led to erratic stock levels [26]. Further assessment of factors such as delivery and transport systems, varied demand for services, human resources capacity, storage space, buffer stocks, and stock management information systems may lead to identification of root causes and solutions [27].

This study has several strengths and limitations. Only 79% of South African public health facilities were contactable. Poorly functioning facilities may be less likely to be contactable and more likely to have stock-outs leading to underestimation of stock-outs. A strength of the survey was the high response rate of contactable facilities. While pharmacists were the preferred respondent, there was a high proportion of nurses-in-charge and nurses, who might be less aware of stock-outs. In turn, non-clinicians like pharmacists might be less aware than clinicians of prescriptions changes in response to stock-outs. Respondents may also be less likely to recall stock-outs which happened in the more distant past than those which are current. These limitations might lead to underestimation of the number of stock-outs. Similarly, individual recall of duration of stock-outs is prone to error and should be treated with caution. Finally, this survey is not able to provide the number of patients leaving facilities without medication.

In May 2016, WHO member states passed a resolution at the World Health Assembly to address medicine shortages with specific requests to the Director General to assess the magnitude and nature of the problem, recognizing the urgency of the situation [28]. This study contributes to address this knowledge gap and can be replicated in other countries and for other essential medicines. Regular monitoring will eventually be necessary to replace ad-hoc studies and target national level interventions for improvement of supply chain systems.

While South Africa has made great strides to extend access to ART as well as increase the quality of the services it provides patients, stock-outs hamper these efforts. The introduction of universal test and treat for HIV means that an increasing number of people need uninterrupted supplies of chronic medication. Differentiated models of care through the dispensing and distribution of medical supplies outside health facilities reduce congestion while also providing patients with more options for prescription refills closer to their homes [29]. However, these models require an uninterrupted supply of medicines. A strong but flexible supply chain system, with regular last mile delivery and robust data management systems is a prerequisite to make this happen. Currently, there are some initiatives underway in South Africa that attempt to address medication stock-outs. The Visibility & Analytics Network (VAN) operating model for coordinated implementation of supply chain interventions has been adopted by the South African NDoH [30] to improve end to end visibility of multiple sources of data and standardize indicators to perform analytics on the supply chain. Other initiatives by NDoH include the new implementation of the Stock Visibility System (SVS), a mobile enabled application used by health facilities to inform the system on supply, and the implementation of a centralized chronic dispensing model whereby medicines are dispensed centrally at the provincial level rather than at a health facility, and then picked up pre-packaged by patients at the health facility [31].

Transparency is essential in a publicly funded supply chain and civilians or their representatives should hold the system accountable and report challenges [18, 32]. Supply crises due to international shortages, health system failure or other reasons, will continue to happen and emergency reporting and reactive mechanisms should exist to anticipate and react to these. End-user monitoring of the supply chain by patients and civil society has the potential to increase transparency and complement public sector monitoring systems.

## Supporting information

S1 Appendix Survey questionnaire. (PDF)

S1 Dataset Survey data. (XLSX)

S1 Table Classification of ARV and TB medicines. HIV Medicines: 3TC–Lamivudine; ABC–Abacavir; AZT–Zidovudine; ATV–Atazanavir; ddI–Didanosine; DRV–Darunavir; d4T –Stavudine; EFV–Efavirenz; FTC–Emtricitabine; FDC–Fixed-Dose Combination of TDF, FTC and EFV; LPV–Lopinavir; NVP– Nevirapine; RTV or /r–Ritonavir; TDF–Tenofovir. TB Related Medicines: IPT–Isoniazid Preventive Therapy; R/H–Rifampicin/Isoniazid; RHZE–Rifampicin/Isoniazid /Pyrazinamide/Ethambutol; E–Ethambutol; ETO–Ethionamide; INH–Isoniazid (for preventive therapy); Km–Kanamycin; Lvx–Levofloxacin; R–Rifampicin; Z–Pyrazinamide; Vit B6 –Vitamin B6 or pyridoxine. * Medicines prescribed as first-choice treatment for the large majority of the patient cohort with no demonstrated resistance to ARVs, were classified as “first-line ARVs.” ** ARVs prescribed for the majority of patients who demonstrated resistance to first-line ARVs were classified as “second-line ARVs”. *** ARVs used for patients who experienced side-effects or resistance to the most frequently used first- and second-line treatment were classified as “ARVs for exceptional cases”. **** All medicines for which the formulation and/or dosage has been adapted for administration to children were classified as paediatric ARVs. These adaptions allow for the variation in children’s weights and ability to swallow pills. ***** For this analysis, only NVP solution for children was classified as an ARV for PMTCT, as it is primarily used in infants of HIV-positive mothers.
(DOCX)
