# Supplementary material for: Stock-outs of antiretroviral and tuberculosis medicines in South Africa: A national cross-sectional survey
Source: PLoS One. 2019 Mar 12;14(3):e0212405. doi: 10.1371/journal.pone.0212405 (PMC6413937; doi:10.1371/journal.pone.0212405)
Supplement: S1 Appendix — (PDF) [file pone.0212405.s001.pdf]

**At your facility, how many patients do you have on ARVs?**

1. Less than a 1000
2. More than a 1000

**Are any ARV or TB medicines out of stock TODAY?**

1. Yes
2. No

(The following questions repeated for each item reported out of stock TODAY)
**What is the name of the ARV or TB medicine?** (See S1 Table )

**How long has the item been out of stock for?**

1. Less than a week
2. 1 to 4 weeks
3. More than a month

**What are you doing for the majority of patients in the mean time?**

1. Turning them away
2. Referred to another facility
3. Switching their treatment (What did they switch them to?)
4. Same treatment BUT given another strength/dosage
5. Borrowed
6. Received 1 or 2 Out Of 3 Drugs;
7. Other

**Did the majority of patients leave with:**

1. No medication
2. Smaller supply of medication (than would have otherwise been issued)
3. Full supply of medication

**Is there another HIV or TB medicine out of stock today?** (Repeat previous 4 questions for each item out of stock)

1. Yes
2. No

**Now I would like you to think about the past 3 months? Can you think back to - ______________(provide date exactly 3 months ago. Ex. If 15 October today, say 15 July 2015).**

**Have any other HIV or TB medicines been out of stock in the past 3 months?**

1. Yes
2. No

(The following questions repeated for each item reported out of stock in the PREVIOUS 3 MONTHS)
**What is the name of the HIV or TB medicine?** (See S1 Table )

**During which month did the problem start/was it out of stock?**

**How long was the item out of stock for?**

1. Less than a week
2. 1 – 4 weeks
3. More than a month

**When the treatment ran out, what did you do for your patients?**

1. Turning them away or referring to another facility
2. Switching their treatment (What did they switch them to?)
3. Same treatment BUT given another strength/dosage
4. Borrowed
5. Received 1 or 2 Out Of 3 Drugs;
6. Other

**Did the patient leave with:**

1. No medication
2. Smaller supply of medication
3. Month/full supply of medication

**Were there any other ARVs or TB medicine out of stock in the last 3 months?** (Repeat previous 5 questions for each item out of stock)

1. Yes
2. No
