# Supplementary material for: Stock-outs of antiretroviral and tuberculosis medicines in South Africa: A national cross-sectional survey
Source: PLoS One. 2019 Mar 12;14(3):e0212405. doi: 10.1371/journal.pone.0212405 (PMC6413937; doi:10.1371/journal.pone.0212405)
Supplement: S1 Table — HIV Medicines: 3TC–Lamivudine; ABC–Abacavir; AZT–Zidovudine; ATV–Atazanavir; ddI–Didanosine; DRV–Darunavir; d4T –Stavudine; EFV–Efavirenz; FTC–Emtricitabine; FDC–Fixed-Dose Combination of TDF, FTC and EFV; LPV–Lopinavir; NVP– Nevirapine; RTV or /r–Ritonavir; TDF–Tenofovir. TB Related Medicines: IPT–Isoniazid Preventive Therapy; R/H–Rifampicin/Isoniazid; RHZE–Rifampicin/Isoniazid /Pyrazinamide/Ethambutol; E–Ethambutol; ETO–Ethionamide; INH–Isoniazid (for preventive therapy); Km–Kanamycin; Lvx–Levofloxacin; R–Rifampicin; Z–Pyrazinamide; Vit B6 –Vitamin B6 or pyridoxine. * Medicines prescribed as first-choice treatment for the large majority of the patient cohort with no demonstrated resistance to ARVs, were classified as “first-line ARVs.” ** ARVs prescribed for the majority of patients who demonstrated resistance to first-line ARVs were classified as “second-line ARVs”. *** ARVs used for patients who experienced side-effects or resistance to the most frequently used first- and second-line treatment were classified as “ARVs for exceptional cases”. **** All medicines for which the formulation and/or dosage has been adapted for administration to children were classified as paediatric ARVs. These adaptions allow for the variation in children’s weights and ability to swallow pills. ***** For this analysis, only NVP solution for children was classified as an ARV for PMTCT, as it is primarily used in infants of HIV-positive mothers. (DOCX) [file pone.0212405.s003.docx]

| **Adult first-line ARVs*** | **Adult second-line ARVs**** | **Adult ARVs for exceptional cases***** | **Paediatric ARVs****** | **PMTCT for children******* | **TB Related Medicines** |
| --- | --- | --- | --- | --- | --- |
| TDF/FTC/EFV 300/200/600 mg, FDC tablets  TDF/FTC 300/200 mg, tablets  3TC 150 mg or 300 mg, tablets  EFV 600 mg, tablets  TDF 300 mg, tablets | AZT 300 mg, tablets  LPV/r 200/50 mg, tablets  ATV 300 mg, tablets  ddI 400 mg, tablets  RTV 100 mg, tablets | NVP 200 mg, tablets  ABC 600 mg, tablets  ABC/3TC 600/300 mg, tablets  DRV 600 mg, tablets  AZT/3TC 300/150 mg, tablets  d4T 30 mg, tablets | ABC 60 mg, tablets  or 20 mg/ml, solution  AZT 100 mg, tablets  RTV 80 mg/ml, solution  3TC 10 mg/ml, solution  LPV/r 80/20 mg/ml, solution  LPV/r 100/25 mg, tablets  AZT 50 mg/5 ml, solution  d4T 15 mg or 20 mg, tablets  EFV 50 mg or 200 mg, tablets | NVP 50 mg/50 ml, solution | R/H 150/75 mg, tablets  R/H 300/150 mg, tablets  R/H 60/60 mg, tablets  RHZE 150/75/400/275 mg, tablets  Z 150 mg or 400 mg, tablets  R 150 mg or 300 mg, tablets  E 100 mg or 400 mg, tablets  ETO 250 mg, tablets  Km 500 mg/2 ml, injection  Lvx 250 mg or 500 mg, tablets  R/H 10 mg/ml, suspension  INH 300 mg, tablets  INH 100 mg, tablets  PN/Vit B6 25 mg or 50 mg, tablets |
